# Perceptual influence of elementary three-dimensional geometry: (1) objectness

**DOI:** 10.3389/fpsyg.2015.01317

**Published:** 2015-08-28

**Authors:** Florentin Wörgötter, Rahel M. Sutterlütti, Minija Tamosiunaite

**Affiliations:** ^1^Faculty of Physics – Biophysics and Bernstein Center for Computational Neuroscience, Georg-August-Universität GöttingenGöttingen, Germany; ^2^Department of Informatics, Vytautas Magnus UniversityKaunas, Lithuania

**Keywords:** object goodness, visio-haptic assessment, 3D-perception, 3D-action, concave-convex

## Abstract

Commonly complex cognitive concepts cannot consistently be connected to simple features of the world. Geometrical shape parameters and (e.g., edge features, compactness, color) may play a role for defining individual objects, but might be too variable to allow for concept formation. Earlier works had suggested that the formation of object concepts is strongly influenced by the division of our world along convex to concave surface transitions. In this first paper in a sequence of two we address this issue using abstract 3D geometrical structures (polycubes). In a first experiment, we let our subjects manipulate and compare polycubes with different compactness and different concavity/convexity asking which of them they would perceive as “an object.” Both parameters (compactness and concavity/convexity) are not correlated in these stimuli. Nonetheless, we find that subjects with clear prevalence choose compact and convex ones. We continue to ask how strongly this influences the way we construct objects. Thus, in a second experiment we let humans combine polycubes to form an object. Also here we find that they prefer compact and convex configurations. This suggests that this simple geometric feature may underlie our cognitive understanding of objectness not only with respect to perception but also by influencing how we build our world.

## 1. Introduction

Gestalt laws, relying on shape parameters and their relations, for example edge-relations, compactness, or others, seem to play a role (Spelke et al., 1993) for forming the concept of an object. For example, Needham and Ormsbee (2003) state that abrupt changes in surfaces featural properties are indications of an object boundary. Color, shapes, and patterns on object surfaces are referred to as featural properties. They could also be defined as the local information in the parts of an object (Schwaninger et al., 2002). Kaufman and Needham (2010) found that 4 month old infants rely on shape, but not color and pattern information to determine the composition of a display. On the one hand, this shows the importance of shape information in processing scenes. On the other hand, this mirrors the slow development of infants ability to see colors which is not fully developed until they are 4 months old (Kellman and Arterberry, 2007). The ability to use colors as source of information in scene processing takes even longer to develop: At 12 months of age, infants use color information in cognitive tasks to individuate, but not to identify objects (Tremoulet et al., 2000). At 12.5 months of age, infants parse objects independent of their rotation using featural information (Needham and Ormsbee, 2003).

However, most—if not all—of the above discussed Gestalt features are quite variable across different objects and object classes, raising the question which feature(s) could unequivocally be used to define the general concept of “being an object.” This study will address this question and focus on transitions between convex and concave surfaces, which had been suggested to play a role in forming object concepts (Rubin, 1958; Koenderink and van Doorn, 1982; Hoffman and Richards, 1984; Biederman, 1987; Braunstein et al., 1989; Cate and Behrmann, 2010; Bertamini and Wagemans, 2013). Currently, evidence for this however remains to some degree indirect because the older studies have performed experiments only in 2 dimensions. In many such experiments subjects had been asked to draw a line across a 2D-figure at the most likely location where this figure would break into two independent entities (e.g., considered as parts of an object). Different from this, the study presented here will addresses the issue of objectness with a 3D approach, where subjects can actually handle and manipulate true 3D structures, assessing objectness in a visio-haptic way.

Interestingly, as early as 1000 AD the first notions arose that shape/object perception might much rely on convexity. In the first known book on visual science, written by the Arab scholar Alhazen (Ibn al-Haytham), 965 - ca. 1040 AD, he stated that connected convex surfaces lead to the perception of a solid object (“if the body has a convex surface that bulges toward the eye […] then if sight perceives the convexity of the surface it will perceive the body's solidity”; (Sabra, 1989, p. 169). Later on it has been suggested by many studies that the Gestalt principle “convexity” predicts which region of an image is perceived as object (Rubin, 1958; Koenderink and van Doorn, 1982; Hoffman and Richards, 1984; Biederman, 1987; Braunstein et al., 1989; Cate and Behrmann, 2010; Bertamini and Wagemans, 2013). Functional magnetic resonance studies provide additional support indicating that convex shapes are encoded in a privileged fashion by human lateral occipital complex (LOC), a region that has been implicated in object recognition (Kourtzi and Kanwisher, 2001; Haushofer et al., 2008).

Quantitative formulations for this had been put forward, for example by Hoffman and Richards who found that whenever two objects interpenetrate, the tangent planes form a region of concave discontinuity (Hoffman and Richards, 1984). Following from this, borders of object parts (or borders of objects) in a scene are characterized by concave discontinuities of their tangent planes. Hoffman and Richards formulated the Minima Rule which says: *Divide a surface into parts at loci of negative minima of each principal curvature along its associated family of lines of curvature* (Hoffman and Richards, 1984, p. 74). Somewhat simplified, this rule says that most likely a natural division between two objects (or between two parts of a given object) can be made—even for smoothly curved shapes—at the location of the extremum (minimum!) of a concave curve. A more detailed discussion on the ontogeny of objects and their parts much related to this principle is found 10 years later in Schyns and Murphy (1994).

Experiments on humans and chimpanzees show that the visual system seems to be more sensitive to concavities than convexities. Hulleman et al. (2000) found a search asymmetry for concavities and convexities. People can easily detect a concavity among many convex distractors, but this pop-up-effect does not occur in the search of a convexity among concave distractors. Chimpanzees are much more sensitive to changes in concavity than in convexity (Matsuno and Tomonaga, 2007). These experimental results indicate that for the visual system of humans and chimpanzees, concavities carry more information about the object than convexities.

Other experiments show that people parse objects at negative minima of curvature. In a recognition task, people were confronted with a surface of revolution consisting of minima and maxima parts on a computer screen. After a few seconds, they were shown four different parts, two taken from the figure and two which did not belong to the figure. Most people (77%) select the minima part that belongs to the figure, whereas the probability to choose it by chance is 25%. This shows that people remember minima parts of objects better than maxima parts (Braunstein et al., 1989), indicating that the minima parts are crucial for object recognition. Also, if asked to draw part boundaries, most people (81%) feel that they are located at local minima (Braunstein et al., 1989). Researchers from Princeton University, New Jersey conducted a large-scale study in which people were asked to draw part boundaries of (mostly) familiar objects. Most of these part boundaries were located at negative minima of principal curvature (Chen et al., 2009). These findings suggest that we perceive concave seams as borders of objects or object parts.

Does this mean that, if a figure has less concave cusps, it is considered more object-like? This is one of the questions, we are addressing in this study and some indications for this already exist. For example, Li et al. (2009) found that there is an inverse proportionality between the complexity of an unknown object and the probability to be considered an object. This study more strongly focused on differences in object understanding of speakers of classifier vs. mass/count languages but one observation states that all their subjects had rated the probability of being an object similarly. Here, solid, simple forms win over solid, complex forms and non-solid, complex forms.

According to Attneave (1957), the complexity of a figure increases if the number of independent turns grows. Furthermore, when there are stimuli differing in complexity and functionality, people select shape-matches (as opposed to match of substance) more often for complex objects and for objects with shape relevant to function (Prasada et al., 2002). This has found to be independent of language development stages in adults as well as young children (Li et al., 2009).

In spite of these supporting findings, Alhazen's conjecture remains still to some degree problematic. Almost all cited studies focused on perception, specifically, of 2D entities^1^, but it is unknown how strongly convex or concave configurations influence our perception of 3D-object-like entities and our actions when we construct 3D-objects.

To address these issues, we will describe two experiments (Experiments A and B) which report on perception as well as construction of a certain type of quite novel 3D geometrical stimuli: Polycubes.

Apart from studies on mental rotation (Shepard and Metzler, 1971), to our knowledge, polycubes have not been used much in psychophysical studies before. Therefore, we will first describe the properties of these geometrical entities and also define measures for them. Only then we will move on to the specific experiments one after the other providing their specific methods followed by the results we obtained.

## 2. Introducing polycubes

**Figure 1:**
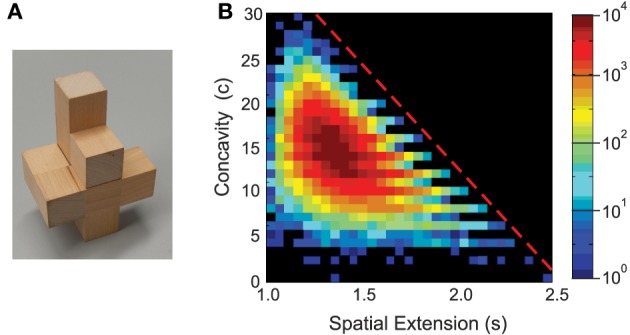
**Characterizing polycubes**. **(A)** Example of a P10 polycube. **(B)** Distribution of concavity vs. spatial extension for all existing (*m* = 346543) P10 polycubes.

Polycubes are obtained when taking any number of regular cubes and attaching them to each other with minimally two surfaces fully touching each other for each pair of cubes (Figure 1A). This way an intriguing, geometrical system is generated with little resemblance to “everyday objects.” There are 29 polycubes existing made from 5 cubes—called P5—and more than 340,000 P10s (Barequet et al., 2009). As an interesting side note we remark that mathematicians so far have not discovered the law that governs the growth rate of the sets of polycubes (*P*1, *P*2, …, *Pn*), *n* ∈ ℕ which seems to be mildly super-exponential in *n* (Aleksandrowicz and Barequet, 2009).

To understand how we use and measure polycubes we need to briefly outline which types of experiments we performed with them (for experimental details see descriptions below).

Experiment A asks to pairwise compare P10 polycubes and quickly select the one which is considered to be “more like an object.”

Experiment B asks to take two P5 polycubes and combine them to a P10 in a way that “an object is being built.”

The conjecture we wish to test is that humans associate objectness to small degrees of concavity. On the other hand, it is known that compactness and orderedness (Garner, 1974; Palmer, 1991; Palmer and Griscom, 2013) are important additional aspects, which strongly influence our assessment of objectness. Thus, in the context of our question these are confounds, which would bias the experiments and against which we must, thus, control.

As a consequence, polycubes need to be described by their three main geometrical properties: (1) spatial extension (the inverse of compactness), (2) concavity, and (3) orderedness, which is strongly correlated to aesthetic appeal (see again Garner, 1974). Indeed, later on we will discuss that very likely there are no other relevant descriptors, which can be seen as 3D-Gestalt principles, for polycubes existing anyhow.

The following paragraphs describe the mathematical aspects of the different measures for spatial extension, concavity, and orderedness of a polycube and can be skipped if not interested in these technical details.

### 2.1. Measures for polycubes

#### 2.1.1. Spatial extension “s”

To calculate this, first the center of mass (CoM) of a P10 is determined. Then one calculates *s* as the sum of the distances of all individual cube-centers to the CoM divided by the number of cubes (i.e., 10). The whole set of all P10s, of which there is a total of 346, 543, yields values of 0.994 ≤ *s* ≤ 2.50.

#### 2.1.2. Concavity “c”

**Figure 2:**
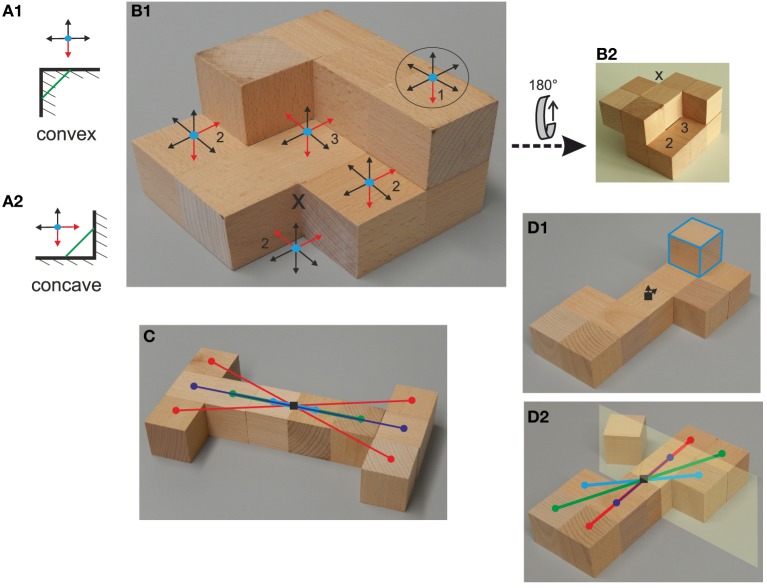
**Defining the measures for concavity and disorder**. **(A,B)** Definition of the concavity index, **(A)** convex and concave configurations in 2D and **(B)** for a real 3D P10. **(B1,B2)** top and bottom view of the same P10. Corresponding location marked by “x” can be used for orientation. **(C,D)** Definition of the disorder index. **(C)** shows an ordered P10. Markers show the *surface projected* Center of Mass (CoM, black square) of the P10 and of every individual cube (colored disks), demonstrating that the blocks appear pairwise- or double-pairwise (red) at equal distances around the CoM making it *ordered*. **(D1)** shows a disordered P10. The CoM is displaced from the center of the central block (indicated by the arrows) and none of the blocks will appear pairwise due to one “unbalancing” block (blue outline). **(D2)** shows a P9 created by removing the unbalancing block from the P10. This P9 is now ordered. All blocks in the P9 except the central block appear pairwise. The central block forms the core of a “central plane” (case 2) around which ordering happens. Hence the disorder index of the P10 in **(D1)** is one (removal of one block has created an ordered polycube).

Figure 2A shows two fundamental rectangular 2D convex and concave constellations, which are conventionally defined in the following way: A connecting line between any two points in a convex configuration (Figure 2A1, green line) will cut through the object, while a similar connecting line in a concave configuration (Figure 2A2) will remain outside. For polycubes such configurations will occur in 3D and only rectangular configurations can exist. Thus, we can equivalently define an index for concavity by a simple counting procedure based on this: Imagine a particle floating above such a surface constellation (Figure 2A, blue disks). The concavity of this constellation is then given by the number of degrees of freedom (DoF) along which this particle *cannot* move freely (indicated by the red arrows in the figure). A convex constellation limits movement only along one DoF (Figures 2A1,B in ellipse), a fundamental concavity limits it along two DoF (Figures 2A2,B, two red arrows), etc. The concavity number *c* is given by the sum of all individual constellations found at the given P10 counting only those that are larger than one (hence disregarding convex constellations). For the example in Figure 2B1 (top-side) we get for the here visible concavities: *c*_*top*_ = 2 + 2 + 2 + 3 = 9. Applying the same counting procedure to the bottom (B2) side we find *c*_*bottom*_ = 2 + 3 = 5 and adding both values we get as total concavity index for this P10 of *c* = 14, which would be the value used for further analyzes. Because participants had to hold all Polycube-objects in their hands, we did not consider concavities against support (e.g., concavities that are arise when putting the object down on a table). This aspect would more concern the problem of how to separate an object from another object (the table) and is less related to the question of objectness as such. For the complete set of P10s we, thus, find 0 ≤ *c* ≤ 30. Other more complex ways of measuring this would be possible, too, but are not required for this study.

Figure 1B shows the joint distribution of concavity plotted against spatial extension for all P10 polycubes. These two parameters are essentially not correlated apart from a diagonal cut-off (dashed line). In particular, there are many polycubes equally compact (low values of *s*) but with widely varying concavity, which is essential for our experiments as discussed below.

#### 2.1.3. Orderedness—defining a “disorder index”

Orderedness of a polycube is strongly correlated with its aesthetic appeal (Garner, 1974; Palmer, 1991; Palmer and Griscom, 2013), which can be assessed using measures based, for example, on Kolmogorow Complexity (Rigau et al., 2008; Schmidthuber, 2010) or other measures (Filonik and Baur, 2009). For example orderedness is a necessary condition for symmetry (Rigau et al., 2008; Schmidthuber, 2010). Hence removing ordered polycubes from the analysis eliminates confounding the results with such aspects and therefore we used only disordered polycubes in all experiments.

How disordered a polycube is is defined by determining how many of its cubes minimally need to be removed until all remaining cubes form an ordered polycube. The number of cubes that need to be removed to achieve this is called the *disorder index*.

We start with the P10. As for spatial extension, we calculate the distances of all individual cube-centers to the CoM. Two cases need to be distinguished: (1) Pair-wise ordering and (2) plane-centric ordering.

1. Pair wise ordering is shown in Figure 2C: If all distances of cubes to the CoM appear pairwise (or multiple pairwise: 4, 6, 8) then this P10 is ordered (and its disorder index is zero). Even numbered polycubes (P10, P8, etc.) can obey this rule, odd ones not.
2. Plane-centric ordering is defined by an “if-and” condition: *If* the individual CoMs of all pairwise ordered cubes fall on onto the same plane *and* all other cubes actually lie on this plane, then this polycube is plane-centric ordered (Figure 2D2). To understand this just imagine a plane formed by some cubes with additional pairwise symmetrical cubes sticking out sideways from this plane (think of a plane “with ears”). Note, the central “plane” can consist also of just one single cube. Even and odd numbered polycubes can obey this rule.

If the P10 is found to be not ordered (hence, cases 1 and 2 do not hold, Figure 2D1) then every single one border cube is removed one after the other asking again, if any of the maximally 10 resulting P9s is now ordered (defined as above). Border cubes are those that can be removed without breaking the structure into two pieces. If, by taking one cube off, an ordered P9 can be made from the original P10, then the disorder index of the original P10 is 1 (Figures 2D1,D2). Otherwise we remove any possible combination of two border cubes (getting P8s), check again and so on.

In general we observed that two cubes added asymmetrically to an ordered P8 (hence getting a P10 with disorder index 2) effectively destroys prettiness or aesthetic appeal of the P10. The resulting P10s look disordered and not nice anymore. Clearly there is no rigorous, overall-agreed measure for this, in which we are not interested here anyhow as this way of ruling out aesthetic appeal is sufficient for the purpose of this study. Thus, in all our analyses, we only consider P10s with a disorder index of 2 or more and thereby we can efficiently eliminate the factor of aesthetics.

## 3. Experiment a—assessment of polycubes

Here we measure how humans perceive abstract 3D structures (polycubes) as objects. The central role of Experiment A is to serve as a large scale control for Experiment B. Other than that Experiment A largely confirms existing data from older 2D studies.

### 3.1. Exp. a—methods

#### 3.1.1. Participants

Participants were 108 healthy adults (age: 22–57) the purpose of this study had not been revealed to them but all experimental procedures had been clearly explained. Participants only partook in the experiment after having given their explicit consent. The experiment is not harmful and no sensitive data had been recorded and experimental data has been treated anonymously and only the instructions explained below (“Procedures”) had been given to the participants. The experiment was performed in accordance with the ethical standards laid down by the 1964 Declaration of Helsinki. We followed the relevant guidelines of the Germany Psychological Society according to which this experiment, given the conditions explained above, does not need explicit approval by an Ethics Committee (Document: 28.09.2004 DPG: “Revision der auf die Forschung bezogenen ethischen Richtlinien”).

#### 3.1.2. Procedures

We asked the participants to perform pairwise comparisons of 10-cube polycubes. To rule out aspects of orderedness all P10 polycubes had a disorder index of minimally 2 and we measured the influence of spatial extension and concavity.

For every comparison, two P10s were given to a subject and they were allowed to manipulate (e.g., turn) them. Care was taken to randomly give objects to the left and right hands of our participants to avoid any handedness-induced biases. We asked our subjects to, please, decide in about 5 s: *Which of the two shown elements corresponds better to your idea of an object?* and recorded their selections. No other instructions were given and their selections were recorded. Every trial took about 30–45 s allowing for a large cohort.

**Figure 3:**
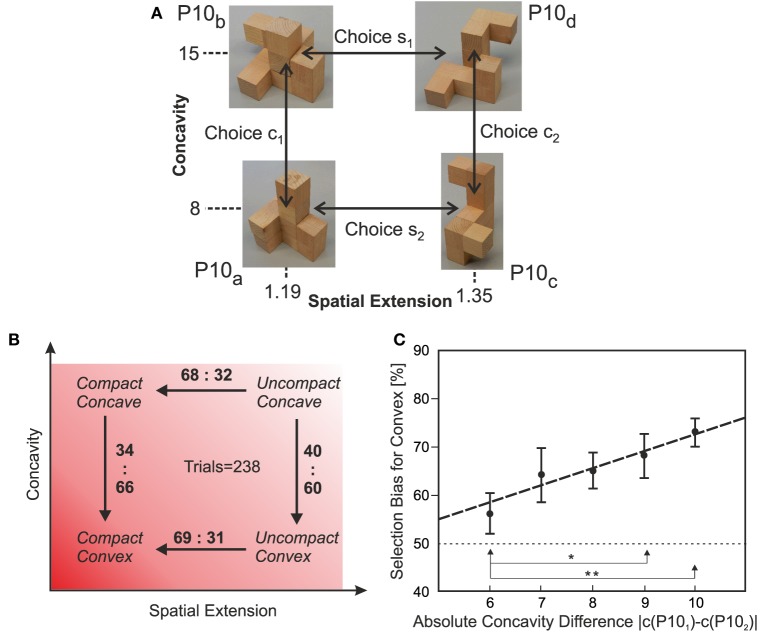
**Comparing pairs of polycubes**. **(A)** Example of one quadruple of P10s used in this study with choice-alternatives indicated. **(B)** Schematic diagram averaging the results for *n* = 238 trials of 4-pair comparisons each, where some had performed more than one experiment rendering 952 pair comparisons in total. Numbers give percent values for the selection bias, which differ significantly from chance (*z*-test, *p* < 0.001). **(C)** Selection bias depends on the differences in concavity. If pairs of polycubes with small differences in concavity are compared then selection is almost random (50%), but increases for larger differences almost linearly. Differences are significant as indicated: ^*^p < 0.05, ^**^p < 0.01 (*z*-test).

The experiment is divided in two parts. For the first part we were investigating the influence of both, spatial extension and concavity. As shown in (Figure 3A) we designed quadruples *P*10_{*a, b, c, d*}_ with two alternating values for *s* and *c*, each: *P*10_*a*_ = (*s*_0_, *c*_0_), *P*10_*b*_ = (*s*_0_, *c*_1_), *P*10_*c*_ = (*s*_1_, *c*_0_), *P*10_*d*_ = (*s*_1_, *c*_1_). Eight quadruples, with different sets of values for *s* and *c*, were used in total.

One trial consisted of four pair-comparisons: *P*10_*a*_ *vs*. *P*10_*b*_, *P*10_*c*_ *vs*. *P*10_*d*_, *P*10_*a*_ *vs*. *P*10_*c*_, and *P*10_*b*_ *vs*. *P*10_*d*_ (Figure 3A). Hence, for every comparison one variable—either spatial extension (compactness) or concavity—was constant.

As all subjects were unpaid volunteers, we asked them to perform only a few trials each (on average 2–3 trials) and data from a total of 238 trials were this way obtained, rendering 238 × 2 = 476 pair comparisons for polycubes of different spatial extension (same concavity) and the same number of comparisons of pairs with different concavity (same spatial extension). All in all in this part of the experiment 952 pairwise P10-comparisons were performed.

In the second part of this experiment we made a deeper investigation into the influence of concavity, by presenting subjects with pairs of P10 polycubes where the spatial extension *s* was the same for a given pair, but using pairs with increasing absolute difference in concavity Δ_*c*_ = | *c*(*P*10_1_) − *c*(*P*10_2_) |. We used Δ_*c*_ = 6, 7, 8, 9, and 10.

For this part of the experiment, we re-used all 476 pair comparisons with different concavity from the first part but we added another 105 pair comparisons to obtain more participant decisions on pairs with bigger differences in concavity (Δ_*c*_ = 9 or 10) for which not enough data existed from above. Note, these additional 105 comparisons were not included into the first part of the experiment, because quadruples do not exist for large differences in concavity, due to the triangular shape of the joint distribution of concavity vs. spatial extension (Figure 1B). Hence polycube pairs for the additional 105 data had to be taken from the left (the “spatially non-extended”) side of the distribution.

#### 3.1.3. Data acquisition, analysis, and statistical tests

In the first part of Experiment A for each pair of polycubes we have recorded the choices, indicated in Figure 3A, made by our participants. From all trials, the total number of selections for concave vs. convex (pairs: *P*10_*a*_ vs. *P*10_*b*_ and *P*10_*c*_ vs. *P*10_*d*_, Figure 3A) as well as compact vs. extended (pairs: *P*10_*a*_ vs. *P*10_*c*_, and *P*10_*b*_ vs. *P*10_*d*_) were counted combining results for all eight used quadruples. Next, proportions were calculated for choosing a more compact object for the respective polycube pairs. The same was done for choosing a more convex polycube. A one-sample single-tailed *z*-test for proportions was performed to check the hypothesis that these proportions are significantly different from 0.5, thereby checking if statistically significant preferences for compact and convex configurations had been obtained.

For the second part of Experiment A we re-grouped our data, considering only those pairs used for investigating the influence of concavity. As this data had not been enough we added 105 additional comparisons as described above. We tested the hypothesis that the preference for convex configurations grows when the difference in concavity between the two polycubes of a given pair Δ_*c*_ = | *c*(*P*10_*a, c*_) − *c*(*P*10_*b, d*_) | increases. Specifically, we formed five sets of pairs with Δ_*c*_ = {6, 7, 8, 9, 10}. For this we had in total {4, 4, 4, 4, 3} sets. Results are plotted as average and standard deviation percentage for convex configuration preference of each set. We checked statistically the hypothesis that with increasing concavity difference the preference for a compact object increases. For this, we performed several two-sample single-tailed *z*-tests for proportions for the different data points (see Figure 3C).

### 3.2. Exp. a—results

Different from previous studies (Rubin, 1958; Koenderink and van Doorn, 1982; Hoffman and Richards, 1984; Biederman, 1987; Braunstein et al., 1989; Cate and Behrmann, 2010; Bertamini and Wagemans, 2013), we use a 3D visuo-haptic approach for perceptual assessment (Exp. A as well as construction, Exp. B, see below) letting our subjects manipulate the experimental objects.

In Experiment A we are assessing how we *perceive* abstract unordered three-dimensional structures (P10-polycubes) as objects. Their shapes appear unstructured and cannot easily be associated to any familiar object, let alone to simple geometrical elements.

We compared the influence of spatial extension *s* and concavity *c* of these structures. We point out one more time that these two parameters are essentially not correlated (Figure 1B). Hence no selection bias is introduced this way.

Using several sets of P10s, we let subjects view and manipulate them asking, which would better correspond to their idea of being an object (see Methods). This was done in several two-alternative force choice experiments using P10-pairs which are either equally concave or equally compact (choices as in Figure 3A). Note the examples in Figure 3A are quite representative for the different P10s that we had used. Subjects, when asked afterwards, were roughly aware about possible differences in compactness, but that there are also differences in concavity remained opaque to them.

Individual selection biases are shown in Figure 3B and are in all cases highly significant (*z*-test, *p* < 0.001). Averaging across all data yields 60–66% preference for convex structures (Figure 3B) and the preference for compact structures is only insignificantly higher (68–69%).

To better quantify this, we looked at the selection bias when considering pairs of polycubes with *differently large differences* in concavity. A central observation is that the selection bias increases from 56% to close to 75% for convex structures if the difference in concavity between both P10s to chose from increases (Figure 3C).

Note, this experiment essentially confirms prior notions about the importance of convexity for objectness, here extended to 3D structure (Rubin, 1958; Koenderink and van Doorn, 1982; Hoffman and Richards, 1984; Biederman, 1987; Braunstein et al., 1989; Cate and Behrmann, 2010; Bertamini and Wagemans, 2013). More importantly, however, Experiment A has been performed as a large scale control for the *Construction* experiment (Exp. B, described next), because in the *Assessment* experiment we did test whether subjects prefer convex constellations even if both P10s have the *same* spatial extension.

## 4. Experiment b—constructing polycubes

This is one core experiment of the current study and addresses for the first time to what degree concavity/convexity influences the way we build abstract objects, for which we again use our polycubes.

### 4.1. Exp. b—methods

#### 4.1.1. Participants and procedures

Participants of these experiments were 18 healthy adults (age: 24–52), the purpose of this study had not been revealed to them their consent obtained and only the instructions mentioned here were given. Other than that the same conditions and, thus, ethical considerations, apply as for Experiment A

For *Construction* we asked our subjects to combine two 5-cube polycubes (P5), to form one P10, by joining them along minimally one contact surface. To not constrain the possible combinatorics we used only those P5s for which more than 500 P10-combinations are possible and for which a wide joint distribution, similar to Figure 1B, for concavity/spatial extension is observed.

Only 18 people participated, because these experiments take about 20–50 min each. Subjects were holding the P5s in their hands and they did not have to put the constructed P10 down. Hence, resulting stability (or not) was not an issue. The instruction given was: *Please combine these two elements in a way that the resulting structure for you best represents an object*. No other instructions were given. The constructed P10 was photographed for later analysis, put to the side and two more of the same P5s were given to the subject. This was repeated for all sets of P5s between 5 and 15 times, depending on the time our subjects wanted to do this, to yield a total of 444 P10s built by all subjects taken together. It could happen that subjects would build the same polycube more than once and we did not prevent this, because we did not want to introduce a bias in their building actions.

#### 4.1.2. Data analysis and statistical tests

Each polycube built by a participant in this experiment was photographed and later entered by hand into a computer as a 3D-matrix of zeros and ones. These matrices were used to calculate spatial extension *s* and concavity *c* of the built polycubes.

**Figure 4:**
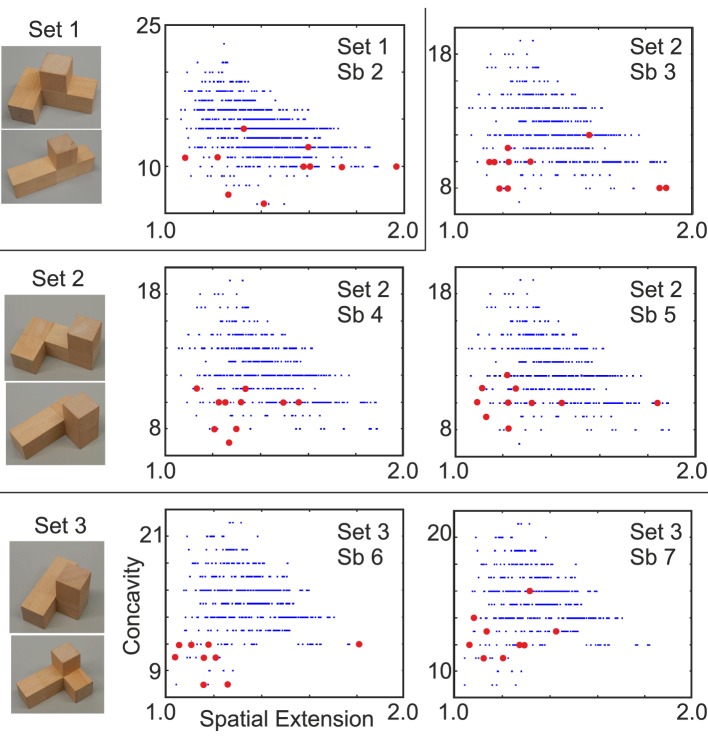
**Six representative examples for the building experiment (Experiment B, polycube sets 1–3)**. Data points obtained from P10s constructed by 6 subjects (Sb) are marked in red. If less than 10 data points are shown then this means that the same polycube(s) had been built several times. Only one result from set 1 is shown because a second example is found in Figure 5.

Furthermore, for every pair of P5 polycubes used in the building experiments we created on a computer all possible (“build-able”) P10s in the same matrix format and calculated *s*- and *c*-values for all of them. This constitutes the basic (joint) distribution within which the actually built P10s form a small subset. Thus, for reference we are plotting these joint distributions (see e.g., Figure 4, small blue dots) and one top in red the (*s, c*) data of the actually constructed P10s.

**Figure 5:**
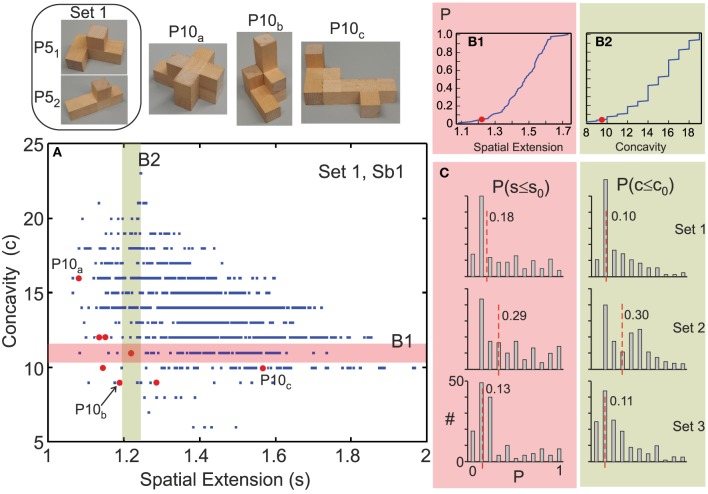
**Subjects prefer compact and convex configurations when constructing polycubes**. **(A)** Scatter plot of the joint distribution of concavity vs. spatial extension for all possible P10s that can be built from one set of P5s (set 1: top, left) and 10 data points from the P10s actually constructed by one subject (Sb 1, note only 8 data points are visible as 2 P10s had been built twice). **(B)** Accumulated probability function calculated along the color bars in **(A)** for **(B1):** *P*(*c* ≤ *c*_0_) and **(B2):** *P*(*s* ≤ *s*_0_) for the red data point in the cross-section of both color bars. For *P*(*c* ≤ *c*_0_), data has been binned between *s*_0_ − 0.025 and *s*_0_ + 0.025 (width of the green bar). **(C)** Histograms of all *P*-values with median indicated (red dashed line) for three sets of P5s (18 subjects, 444 P10s built in total).

For statistical analysis, we treated spatial extension *s* and concavity *c* as two independent random variables and calculated the accumulated probability for obtaining data points less or equal to the actually built ones (*s*_0_, *c*_0_) along both axes as: *P*(*c* ≤ *c*_0_) and *P*(*s* ≤ *s*_0_) (Figures 5B1,B2). For *P*(*c* ≤ *c*_0_), data has been binned between *s*_0_ − 0.025 and *s*_0_ + 0.025. The obtained cumulative probabilities, tell how (un-)likely it is to build the respective P10 by a random process^2^.

The probabilities *P*(*c* ≤ *c*_0_) and *P*(*s* ≤ *s*_0_) for the same P5-polycube pair were collected for all subjects and all trials performed and plotted as histograms (Figure 5C). Finally medians were calculated, which are descriptive for the choice-trends of our participants.

### 4.2. Exp. b—results

When constructing P10 polycubes from two P5s our subjects also preferred with high significance convex and compact configurations. Note, in this experiment we only used P5s which, when combining, resulted in wide P10-distributions of spatial extension (*s*) vs. concavity (*c*) with only unordered P10s. By this a large range for building was available and an aesthetic bias was avoided. Characteristic joint distributions of spatial extension vs. concavity for all possible combinations, which can be constructed from one pair of P5s are show in Figure 4 and, indeed, cover rather wide ranges. In spite of this, subjects would usually construct P10s with values in the lower left quadrant of such a distribution (see red dots in Figures 4, 5A). In Figure 5A we also show three example of P10s built by “Subject 1” (panel A, top).

As described above, we plotted histograms of the accumulated probability for obtaining data points less or equal to the actually built ones, hence for *P*(*c* ≤ *c*_0_) and *P*(*s* ≤ *s*_0_) (Figures 5B1,B2). These histograms have very low medians (Figure 5C) and are visibly skewed to the left demonstrating a far from chance prevalence for compact and convex configurations with few outliers.

**Figure 6:**
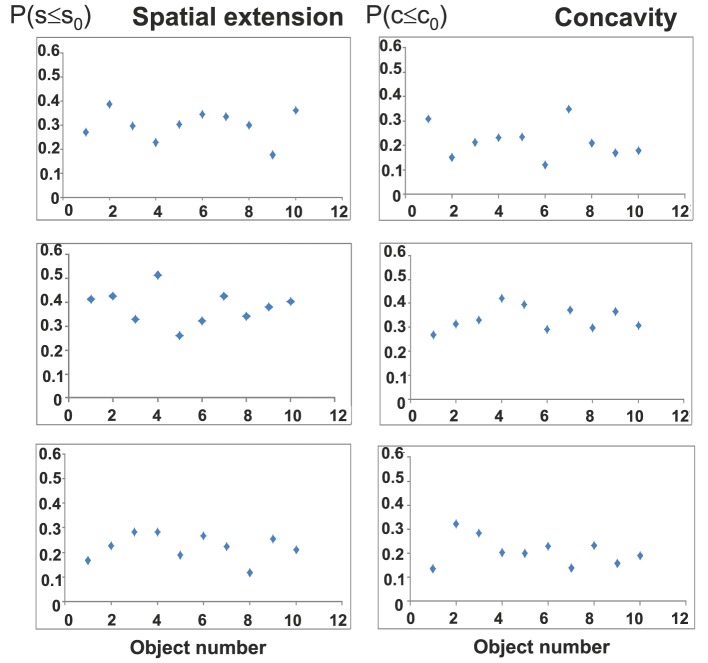
**There is no parameter tendency visible when polycubes are built one after the other**. Shown is the development of the *P*(*s* ≤ *s*_0_) and *P*(*c* ≤ *c*_0_) values in the course of some experiments (object number corresponds to experimental sequence).

Figure 6 shows by ways of six examples that there is no tendency visible from our subjects toward consecutively building more (or less) convex or compact objects. This holds true for the whole cohort.

## 5. Discussion

The hypothesis that concave-convex surface transitions are instrumental for our object understanding is an old one and there are several individual lines of evidence from perception that are supporting this (Rubin, 1958; Koenderink and van Doorn, 1982; Hoffman and Richards, 1984; Biederman, 1987; Braunstein et al., 1989; Cate and Behrmann, 2010; Bertamini and Wagemans, 2013). Naturally, perception (visual and haptic) also underlies our approach in each of the experiments, but we have—different from the existing studies—coupled it to action (construction) where in both experiments we focus on 3 dimensional entities. We asked: Is there an implicit object understanding existing in adults that is coupled to the feature of convexity?

The main difficulty in addressing this question was that we needed to design a set of stimuli where the relevant parameter (concavity) can be controlled independently of other factors and where there are no easy associations possible to everyday objects. Furthermore, the experimental set needed to allow for perceptual assessment *and* action/construction. Polycubes proved to be an intriguing, geometrical system that allowed us to generate the required set (after removal of potentially interfering aspects of aesthetics). Thus, one major difference from the majority of earlier studies is that here we used 3D visuo-haptic stimuli allowing participants to manipulate them when assessing and/or building “objects.” Almost all other studies that have addressed similar questions were using 2D stimuli, often on computer screens. The use of 2D-stimuli makes it difficult—or impossible—to compare perception (our Exp. A) with aspects were perception is directly coupled to action, like here were participants where asked to build “objects” (Exp. B).

In the first experiment (*Assessment*), we asked people to compare two P10s and to select the one which is more like an object. There was a clear preference toward compact and convex configurations. As mentioned above, we were carefully excluding all ordered (e.g., symmetric) forms in Experiments A and B. The disorder index of each P10 was higher or equal to two, which means that at least two blocks would have to be taken away from the P10 to make it ordered. However, it might be possible that the assured “asymmetry” is appealing to people and that their decisions can still be influenced by aesthetics. McManus (2005) shows that in the arts of the renaissance, slight asymmetry is considered more appealing than complete symmetry. Indeed, we did observe a few subjects (less than 10% of the cohort) who always chose the more complex (the more concave!) P10 in all comparisons. It would certainly be interesting to assess to what degree subjects working in the arts and who are possibly more familiar with abstract and complex 2D- as well as 3D-percepts might show a similar bias, which might confirm an aesthetic influence.

Similarly one could ask when and how the perceptual influence of concave-convex surface transitions might be present in infant/child development. Clear indications exist from many studies that featural changes play an important role for defining object boundaries and that “shape” may be one of the central features (Spelke et al., 1993; Needham and Ormsbee, 2003; Kaufman and Needham, 2010) for forming concepts of objectness already at an infant age. These aspects, however, exceed the scope of the current study.

To our knowledge there are no other studies existing that have tried to address the question of *implicit* human concepts of objectness by monitoring how we build them. This is to some degree intriguing because—different from animals—constructing, molding, shaping, etc. are major traits of humans and, clearly, there are more human-made objects existing than natural ones. Hence there ought to be a connection between “what we perceive as an object” and “what we build so as to be an object.” On the other hand it is clearly difficult to address this issue due to the fact that normal, everyday objects usually always carry some semantics for us. Hence whether or not there is a parameter existing that could be coupled to a raw, implicit concept of objectness when building them would always be confounded by “meaning” (of components as well as of constructive combinations). Thus, we are usually not building “objects as such” but “things with some (functional) meaning.” Gibson's affordance principle (Gibson, 1977) as well as the concept of Object-Action Complexes (OACs, Wörgötter et al., 2009; Krüger et al., 2011) both point in the direction that for us objects always carry semantics. Due to this we needed to find a way to eliminate semantics in order to test our hypothesis. As discussed above, polycubes provided the solution to this problem. Of importance here is that compactness and convexity are essentially not correlated for the sets of polycubes used for constructions. The joint distributions (Figures 4, 5A) do not differ from that of the complete set (Figure 1B) and there is no correlation-based bias introduced in this experiment.

As for *Assessment* also the *Construction* experiment showed that (most) people have a strong preference for compact and convex configurations. When asked afterwards they told that their choice was determined by a preference for compactness. They were not aware of the strong influence of convexity.

Is it possible that there are other hidden parameters present in the polycubes, which might influence decisions? We do not think so. Apart from aspects of order and symmetry (aesthetics), we feel hard pressed to come up with anything here. Polycubes almost never resemble anything we know and it seems unlikely that there are more variables that are relevant for this study existing in this system.

### 5.1. Conclusion

This study confirms that objects seem to receive their “objectness” very much from convexity. This aspect is strongly active in 3D and it influences also the way by which we construct “an object,” which is one major novel finding of this work. This study is now followed by a second paper (Tamosiunaite et al., under review) that addresses the question how objects break into parts based on the same convex-concave surface transitions as investigated here. The second study strongly focuses on the aspect to what degree these parts may carry for us “meaning” and we will show that convex-concave surface transitions will lead to parts that can be named, which can be seen as another strong indicator for the perceptual power of this low-level feature.

## Funding

The research leading to these results has received funding from the European Community's Seventh Framework Programme FP7/2007-2013 (Specific Programme Cooperation, Theme 3, Information and Communication Technologies) under grant agreement no. 270273, Xperience.

### Conflict of interest statement

The authors declare that the research was conducted in the absence of any commercial or financial relationships that could be construed as a potential conflict of interest.

## Footnotes

^1^There is an additional level of complexity arising when considering 2D entities. They can be problematic as the distinction between “inside” and “outside,” hence the viewpoint of the observer, may turn concavity and convexity around. Usually one uses closed 2D forms and naively assumes the outside-viewpoint. These problems do not exist in 3D.

^2^*P*(*X* ≤ *x*) defines the cumulative probability given by $\int_{-\infty }^{x}f_{X}(t)dt$ with *f*_*X*_ the probability density function for the random variable *X*. P must not be confused with p, the confidence threshold of a statistical test.
